# Genetic Transformation of Apomictic Grasses: Progress and Constraints

**DOI:** 10.3389/fpls.2021.768393

**Published:** 2021-11-05

**Authors:** Andrés M. Bellido, Eduado D. Souza Canadá, Hugo R. Permingeat, Viviana Echenique

**Affiliations:** ^1^Departamento de Agronomía, Centro de Recursos Naturales Renovables de la Zona Semiárida (CERZOS – CCT – CONICET Bahía Blanca), Universidad Nacional del Sur (UNS), Bahía Blanca, Argentina; ^2^AGROBIOTEC-FCA, Facultad de Ciencias Agrarias UNR, Santa Fe, Argentina

**Keywords:** genetic transformation, apomictic grasses, plant regeneration, DNA-delivery methods, editing, morphogenic regulators

## Abstract

The available methods for plant transformation and expansion beyond its limits remain especially critical for crop improvement. For grass species, this is even more critical, mainly due to drawbacks in *in vitro* regeneration. Despite the existence of many protocols in grasses to achieve genetic transformation through *Agrobacterium* or biolistic gene delivery, their efficiencies are genotype-dependent and still very low due to the recalcitrance of these species to *in vitro* regeneration. Many plant transformation facilities for cereals and other important crops may be found around the world in universities and enterprises, but this is not the case for apomictic species, many of which are C4 grasses. Moreover, apomixis (asexual reproduction by seeds) represents an additional constraint for breeding. However, the transformation of an apomictic clone is an attractive strategy, as the transgene is immediately fixed in a highly adapted genetic background, capable of large-scale clonal propagation. With the exception of some species like *Brachiaria brizantha* which is planted in approximately 100 M ha in Brazil, apomixis is almost non-present in economically important crops. However, as it is sometimes present in their wild relatives, the main goal is to transfer this trait to crops to fix heterosis. Until now this has been a difficult task, mainly because many aspects of apomixis are unknown. Over the last few years, many candidate genes have been identified and attempts have been made to characterize them functionally in *Arabidopsis* and rice. However, functional analysis in true apomictic species lags far behind, mainly due to the complexity of its genomes, of the trait itself, and the lack of efficient genetic transformation protocols. In this study, we review the current status of the *in vitro* culture and genetic transformation methods focusing on apomictic grasses, and the prospects for the application of new tools assayed in other related species, with two aims: to pave the way for discovering the molecular pathways involved in apomixis and to develop new capacities for breeding purposes because many of these grasses are important forage or biofuel resources.

## Introduction

Apomixis in plants is defined as an asexual type of reproduction by seeds where the maternal plant produces clonal offsprings, combining the advantages of propagation by seeds and those of propagation by clone (Kandemir and Saygili, 2015). This reproductive mode, although rare among the major crop families, is a common trait in the polyploid C4 grasses (Poaceae family), many of which are important forage species. Some genera included in this group are *Panicum, Pennisetum, Urochloa(Brachiaria), Eragrostis, Tripsacum, Paspalum, Cenchrus, Brachipodium*, *Bothriochloa, Bouteloua, Callipedium*, *Dischantium, Hyparrhenia, Melinis*, *Setaria, Chloris* (Quero-Carrillo et al., 2010). For many years our group has been working on deciphering the genetic pathways involved in apomixis in *Eragrostiscurvula* or “weeping lovegrass,” a facultative apomictic grass native to Southern Africa and well-adapted to grow in EEUU, Australia, and the semiarid regions of Argentina. We recently demonstrated that apomixis in this grass is a gene-regulated trait linked to a chromosome region inherited in a Mendelian way (Zappacosta et al., 2019), which is consistent with other previously reported findings (Voigt and Bashaw, 1972). Additionally, we were able to sequence and assemble the genome of “Victoria,” a diploid accession with high resolution (Carballo et al., 2019), which demonstrated a strong epigenetic mechanism controlling the trait (Selva et al., 2020; Carballo et al., 2021). This complex epigenetic landscape makes it essential to analyze the trait directly in an apomictic context.

**FIGURE 1:**
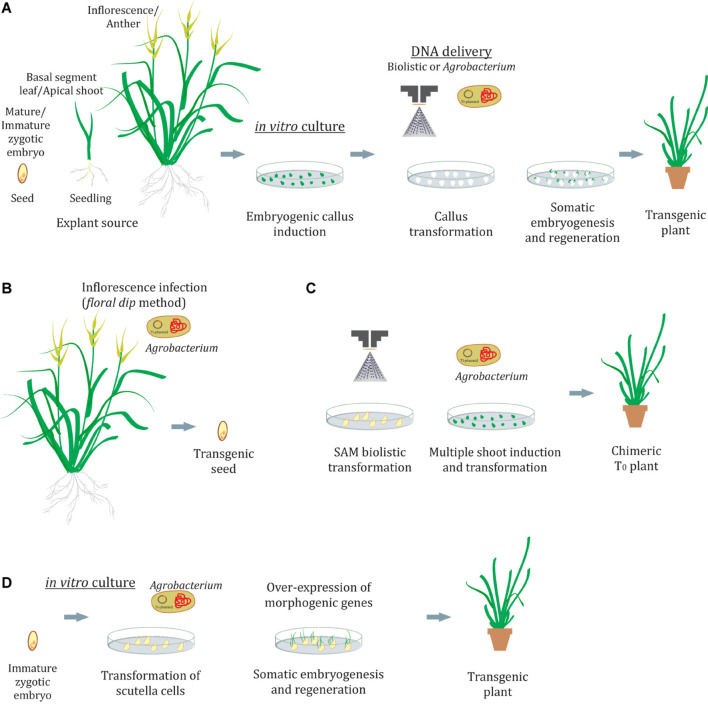
Genetic transformation in grasses. **(A)** Transformation of calli and regeneration by indirect somatic embryogenesis. **(B,C)** *In planta* methods by floral dip **(B)** or shoot apical meristem transformation **(C)**. **(D)** Transformation of scutella cells with morphogenic regulators and regeneration without the callus phase by direct somatic embryogenesis. SAM, shoots apical meristem.

Plants are organisms with a plastic development and as such have the potential to differentiate new organs from the stem cell niche (pluripotency). Also, plant cells can be “reprogrammed” to produce an entirely identical plant (clone) through embryogenesis. This phenomenon is called totipotency and is exploited in biotechnology to induce somatic embryogenesis and produce transgenic plants. In grasses, success in genetic transformation is strongly dependent on the tissue culture procedures and on the ability of plant cells to regenerate a whole plant (Figure 1A). This usually involves the induction of embryogenic callus in a medium supplemented with auxins and the development of embryos in a medium with cytokinins, such as 6-benzylaminopurine (BAP) or thidiazuron (TDZ) or metatopolines at low concentrations (Sánchez-Romero, 2021). Several morphogenic regulators are able to modify calli responsiveness to embryogenesis, transformation, and regeneration (Fehér, 2019; Gordon-Kamm et al., 2019). Also, it is commonly observed that even the genotype and explant source might condition these callus responses (Echenique et al., 1996; Colomba et al., 2006; Yadav et al., 2009; Seo et al., 2010; Cabral et al., 2011; Kumar and Bhat, 2012; Takamori et al., 2015). As genotype effects are unavoidable, different explants, culture conditions, and morphogenic regulators should be assayed to improve the transformation and regeneration efficiencies. Several plant tissues have been used as explants to induce embryogenic calli, such as zygotic embryos, scutella, apical shoots, inflorescences, anthers, and basal parts of the leaves, among others. The actively dividing cells present in the callus phase provide a suitable target for transformation (Delporte et al., 2012).

To achieve a successful genetic transformation not only is it critical to establish a suitable tissue culture system but also determine an effective DNA delivery method. The most commonly used methods in grasses are biolistic- and *Agrobacterium*-mediated transformation. The former, in theory, can be applied to any species or plant tissue owing to its physical character but requires trained personnel and also specialized and expensive instruments such as a gun device (Ozyigit and YucebilgiliKurtoglu, 2020). Furthermore, the DNA delivered by particle bombardment could result in variable expression levels in different transformed clones owing to the random integration of the expression cassettes in the host genome. In addition, if either several integrations of the same gene or integrations in methylated zones occur, it could result in transgene silencing (Delporte et al., 2012; Anami et al., 2013; Dong and Ronald, 2021). Also, the sequences of the expression cassettes introduced are prone to be truncated reducing the transformation efficiency (Shou et al., 2004). On the other hand, *Agrobacterium*-mediated transformation appears to be more suitable since it produces low transgene copy number insertions and confers more stability over generations with reduced gene silencing (Singh and Prasad, 2016). However, as *Agrobacterium*-mediated transformation is a biological process it is still dependent on the interaction of the *Agrobacterium* strain with the host plant genome, which is known to be influenced by several factors. Phenolic compounds produced by the host plant are necessary to induce the expression of *Agrobacterium vir* genes. In monocots, which are not natural hosts and are considered recalcitrant to *Agrobacterium* infection, these compounds are not synthesized and the addition of acetosyringone during plant and bacterial interaction supports the transference of the gene (Jha et al., 2011). Furthermore, 20 min of vacuum treatment and cocultivation for 2–3 days have been necessary to enhance this interaction (Li and Qu, 2011; Lin et al., 2017). Another important issue in plant transformation is the selection agent used to detect and isolate the primary transformed cells. Cells subjected to repetitive stresses related to transformation, antibiotic selection, and the more-or-less long-term *in vitro* cultures are exposed to increased risk of somaclonal variation (Lu et al., 2006; Carloni et al., 2014; Ishigaki et al., 2014). This variation refers to the genetic changes in either the DNA content (ploidy changes) or the DNA structure (chromosome rearrangements and point mutations) that are common in regenerated plants. For example, albinism in regenerated seedlings is usually indicative of a somaclonal event in the chloroplast genome (Mozgova et al., 2012). A reduction of time in culture, inducing direct regeneration of apical shoots without a callus phase, and employing non-antibiotic selection methods (i.e., capacity to metabolize mannose) offer new alternatives to alleviate concerns about somaclonal variation (O’Kennedy et al., 2004; Jha et al., 2011).

Owing to their efficient use of energy and high adaptability to harsh conditions, C4 grasses and their wild relatives constitute a source of genes that can be useful to genetically improve crop plants for agronomic traits such as high yield and stress resistance. Even when the transformation of grasses is a difficult task, this technology has been used to improve forage and turf in different aspects, such as digestibility, drought, cold, freezing, and salt tolerance, virus-resistance, among other traits in species like Bahiagrass, Tall fescue, Switchgrass, and Kentucky bluegrass (Wang and Brummer, 2012). Additionally, there have been many efforts focused on the use of these grasses for the production of biofuels with encouraging progress (Weijde et al., 2013; Mazarei et al., 2020). On the other hand, apomixis is a promising alternative that can provide a direct mechanism for the exploitation of heterosis. This is an agronomically important feature in breeding programs that would dramatically reduce the costs of hybrid seed production, especially for crops like maize, rice, or wheat. Alternatively, the transformation of an apomictic clone is an attractive strategy as the transgene is immediately fixed in a highly adapted genetic background, capable of large-scale clonal propagation. Although not providing complete transgene containment, gene transfer between apomictic species occurs at low frequency and over short distances, as has been shown in Kentucky bluegrass (*Poa pratensis*) and Bahiagrass (*Paspalum notatum*) (Johnson et al., 2006; Sandhu et al., 2010). Many efforts have been taken to understand the nature of apomixis, and several apomictic related genes have been identified by differential expression analyses (reviewed in Hojsgaard and Hörandl, 2019; Kaushal et al., 2019; Schmidt, 2020). Although it was suggested that the asexual mode of reproduction could have evolved from the altered expression of genes in the sexual pathway (Carman, 1997), only partial engineering of apomixis could be obtained by transgenesis in *Arabidopsis thaliana* (Ravi et al., 2008; d’Erfurth et al., 2009; Ravi and Chan, 2010) and rice (Khanday et al., 2019; Wang et al., 2019) due to the complexity of the trait. Although all the above efforts represent significant progress in the possibility of transferring the trait, an understanding of the mechanism that operates in true apomicts is crucial to getting efficient synthetic apomixis. This knowledge could be obtained by altering the genes or genetic pathways involved using genome-editing approaches in tissues or using single cells (protoplasts) in automated platforms (Dlugosz et al., 2016). Automation would enable large-scale screens, such as those performed by Wang et al. (2015), where CRISPR-mediated mutations were used to determine the essential genes required for human cell proliferation. By using an automated cell screen, every gene could be knocked out sequentially in the crop cells for a massive functional analysis (Altpeter et al., 2016). Regardless of the approach selected, this functional analysis and also the exploitation of traits with agronomic relevance present in apomictic grasses is still dependent on genetic transformation and plant regeneration, which are the main bottlenecks in the process. In this study, we review the current status of the *in vitro* culture and genetic transformation methods focusing on apomictic grasses (Table 1), and the prospects for the application of new tools assayed in other related species, with two aims: to pave the way for discovering the molecular pathways involved in apomixis and to develop new capacities for breeding purposes because many of these grasses are important forage or biofuel resources.

**TABLE 1:** Summary of methods for *in vitro* culture, transformation and regeneration of apomictic grasses and their relatives.

Specie	Reproductive mode	Explant source	Callus induction	Transformation method	Transferred genes	Transgenic cells selection	Regeneration	References
*Eragrostis curvula*	Sexual Apomictic	Immature inflorescences	MS medium + 2,4-D + BAP	not evaluated	*−*	*−*	MS medium + NAA + BAP	Echenique et al., 1996
*Eragrostis curvula*	Apomictic	Immature inflorescences, embryo, seed, leaves bases	MS medium + 2,4-D + BAP + caseine hydrolyzates + sucrose	not evaluated	*−*	*−*	MS medium + 2,4-D + caseine hydrolyzates + MS vitamins	Echenique et al., 2001
*Eragrostis curvula*	Apomictic	Leaf, seed	MS medium + 2,4-D + BAP + sucrose	Biolistic	*Hsp12, gus*	GUS-screening	MS medium + 2,4-D + BAP + sucrose	Ncanana et al., 2005
*Eragrostis curvula*	Apomictic	Immature inflorescences	MS medium + 2,4-D + BAP + caseine hydrolyzates + sucrose	Biolistic	*uidA*	GUS-screening	MS medium + sucrose	Diaz, 2006
*Eragrostis tef*	Sexual	Immature embryos	KBP medium + 2,4-D + caseine hydrolyzates + glutamine + MES + maltose	*Agrobacterium* (LBA4404)	*PcGA2ox1, nptII*	Kanamycin (200 mg/L)	K4NB medium + BAP + CuSO_4_ + glutamine + maltose	Gebre et al., 2013
*Panicum virgatum*	Sexual	Inflorescences	MS medium + 2,4-D + BAP; LP9 (NB-based) medium + 2,4-D + L-proline + sucrose + glutamine + caseine hydrolyzates	*Agrobacterium* (EHA105)	*hph pporRFP*	RFP-screening, Hygromicin (60 mg/L)	MS medium + BAP	Burris et al., 2009
*Panicum virgatum*	Sexual	Mature seed	MS medium + 2,4-D + BAP + maltose + L-proline	*Agrobacterium* (EHA105)	*hpt, sGFP*	GFP-screening, Hygromicin (100–200 mg/L)	MS medium + NAA + BAP + GA + maltose	Li and Qu, 2011
*Panicum virgatum, P. maximum, P. longijubatum, P. meyerianum, P. capilare, P. halli, P. stapfianum*	Sexual Apomictic	Immature embryos, shoot apices, mature seeds	MS medium + 2,4-D + sucrose	not evaluated	*−*	*−*	MS medium + NAA + TDZ + maltose	Seo et al., 2010
*Panicum virgatum*	Sexual	Mature seed	MS medium + 2,4-D + sucrose	*Agrobacterium* (EHA105)	*CAD* RNAi	Hygromycin (75 mg/L)	MS medium + Kinetin + sucrose	Fu et al., 2011a, b
*Panicum virgatum*	Sexual	Mature seed	NB medium + 2,4-D + L-proline + maltose	*Agrobacterium* (EHA105)	*hpt, gus*	Hygromycin (50–100 mg/L), GUS-screening	MS medium + NAA + GA + BAP + maltose	Liu et al., 2015
*Panicum virgatum*	Sexual	Mature seed	NB medium + 2,4-D + BAP + L-proline	*Agrobacterium* (EHA105)	*hph, gus*	Hygromycin (50–100 mg/L)	MS medium + BAP + IAA + Kinetin + NAA + L-proline	Lin et al., 2017
*Panicum virgatum*	Sexual	Mature seeds, inflorescences	MS medium + 2,4-D + BAP + L-proline + B5 vitamin + maltose	*Agrobacterium* (GV3101, GV2260, EHA105, GV3850)	*hpt, pporRFP*	RFP-screening	MS medium + NAA + BAP + GA + maltose	Ondzighi-Assoume et al., 2019
*Pennisetum glaucum*	Sexual	Inflorescences	MS medium + 2,4-D + sucrose	Biolistic	*bar, uidA, gfp*	PPT (3–10 mg/L), GUS-, GFP-screening	MS medium + TDZ + BAP	Goldman et al., 2003
*Pennisetum glaucum*	Sexual	Immature embryos	MS medium + 2,4-D + sucrose L3 medium + 2,4-D + L-proline + maltose	Biolistic	*manA*	Mannose	MS medium + IAA + Kinetin + A_*g*_NO_3_	O’Kennedy et al., 2004
*Pennisetum glaucum*	Sexual	Shoot apices	*−*	*Agrobacterium* (EHA105)	*hptII, gus*	Hygromycin (30 mg/L), GUS-screening	MS medium + sucrose	Jha et al., 2011
*Poa pratensis*	Apomictic	Immature embryos	MS medium + 2,4-D + BAP + sucrose	Biolistc	*hpt, gus. bar*	Bialaphos (2 mg/L), Hygromycin (100 mg/L)	MS medium + Kinetin	Gao et al., 2006
*Poa pratensis*	Apomictic	Shoot apices	MS medium + 2,4-D + BAP	*Agrobacterium* (LBA4404)	*betA, als*	Chlorsulfuron (6 mg/L)	MS medium + BAP	Zhang et al., 2010
*Cenchrus ciliaris*	Sexual Apomictic	Mature seeds, shoot apices, immature inflorescences	MS medium + 2,4-D + BAP	not evaluated	*−*	*−*	MS medium + 2,4-D + BAP or Kinetin	Yadav et al., 2009
*Cenchrus ciliaris*	Apomictic	Anthers	MS medium + 2,4-D + sucrose	not evaluated	*−*	*−*	MS medium + BAP + NAA + sucrose	Carloni et al., 2014
*Cenchrus ciliaris*	Apomictic	Shoot apices	*−*	not evaluated	*−*	*−*	MS medium + TDZ + GA	Kumar and Bhat, 2012
*Cenchrus ciliaris*	Apomictic	Immature inflorescences	MS medium + 2,4-D + BAP + sucrose	Biolistc, *Agrobacterium* (EHA105)	*hptII, gus*	GUS-screening, Hygromycin (30 mg/L)	MS medium + 2,4-D + BAP + Caseine hydrolyzate + L-proline + L-arginine	Laishram et al., 2020
*Urochloa ruziziensis*	Sexual	Shoot apices	MS medium + 2,4-D + BAP + sucrose	Biolistic	*gus, bar*	GUS-screening, bialaphos (10–2 mg/L)	MS medium + BAP + NAA	Ishigaki et al., 2009, 2012
*Urochloa brizantha*	Sexual Apomictic	Mature seeds, leaf base segments	MS medium + 2,4-D + BAP + Caseine hydrolyzate	Biolistic	*hptII, gus*	GUS-screening, Hygromycin (5–10–20 mg/L)	MS medium + BAP + NAA + kinetin + Caseine hydrolyzate	Cabral et al., 2011, 2018
*U. brizantha, U. decumbens, U. humidicola, U. ruziziensis*	Apomictic Sexual	Mature seeds	MS medium + 2,4-D or picloram + sucrose + Caseine hydrolyzate	Not evaluated	*−*	*−*	1/2MS medium + BAP	Takamori et al., 2015
*U. brizantha, U. decumbens, U. ruziziensis*	Apomictic Sexual	Leaf base segments	MS medium + 2,4-D or picloram or TDZ + sucrose + proline + hydrolyzed caseine + myo-inositol	Biolistic	*gus*	GUS-screening	1/2MS medium + BAP	Yaguinuma et al., 2018
*Paspalum notatum*	Apomictic	Mature seeds	MS medium + BAP + dicamba + sucrose + CuSO_4_	Biolistic	*nptII, DREB1A, HvWKY38, bar, GA2*	Paromomycin (50 mg/L)	MS medium + BAP + sucrose + CuSO_4_	Altpeter and James, 2005; Agharkar et al., 2007; Sandhu et al., 2007; James et al., 2008; Xiong et al., 2010
*Paspalum notatum*	Sexual	Mature seeds	MS medium + 2,4-D + sucrose	Biolistic	*gus, bar*	GUS-screening, bialaphos (3–10 mg/L)	MS medium + 2,4-D, BAP + CuSO_4_	Gondo et al., 2005
*Paspalum notatum*	Sexual	Mature seeds	MS medium + 2,4-D + sucrose + CuSO_4_	Biolistic	*gfp,bar*	GFP-screening	MS medium + GA_3_ + kinetin	Himuro et al., 2009
*Paspalum notatum*	Apomictic Sexual	Mature seeds	MS medium + B5 vitamins + 2,4-D + sucrose	Biolistic	*egfp, bar, QGJ, TGS1-like*	GFP-screening, ammonium glufosinate (1 mg/L)	MS medium + B5 vitamins + BAP + GA_3_ + CuSO_4_ + sucrose	Mancini et al., 2014, 2018; Colono et al., 2019
*Paspalum dilatatum*	Sexual	Mature seeds, shoots	MS medium + 2,4-D + sucrose	Biolistic	*nptII, PdCCR1*	Paromomycin (50 mg/L)	MS medium + kinetin + sucrose	Giordano et al., 2014

## Apomictic Grasses Transformation Status

### *Eragrostis* Wolf

*Eragrostis curvula* (Schrad.) Nees is a polymorphic grass native to Southern Africa, naturalized in the semiarid regions of Argentina. Lovegrasses were introduced to Australia, the United States, and Argentina as soil conservation cultivars or forage grasses, having important agronomic traits such as drought resistance and a perennial habit, with forage quality being the most limiting factor. Most *E. curvula* cultivars are polyploids and reproduce by diplosporous apomixis. Diploids are always sexual and are very infrequent in nature, with tetraploid apomicts being the most frequently used cultivars for forage. In recent years our group has been working on different aspects of the reproductive biology of this grass, and different genetic and genomic resources have been developed, such as more than 40 floral and leaf transcriptomes (Cervigni et al., 2008a; Garbus et al., 2017), a genome assembly (Carballo et al., 2019), more than 4,520 SSRs (Cervigni et al., 2008b; Carballo et al., 2019), the first linkage map for the species highly saturated with GBS-SNPs (Zappacosta et al., 2019), genes and/or alleles conferring resistance to abiotic stresses (Carballo et al., 2019; Selva et al., 2020), and genes involved in lignin pathways (Díaz et al., 2010; Carballo et al., 2019).

Tissue culture protocols for *E. curvula* have been established using panicles as explant donors obtaining regeneration by embryogenesis and organogenesis (Echenique et al., 1996). Inflorescences just emerging from the flag leaf were cultured on Murashige and Skoog medium (1962) supplemented with different concentrations of 2,4-D and BAP, the most suitable concentrations for calli growth and development being 9 and 18 μM 2,4-D combined with 0.044 μM BAP. Fertile plants were obtained from four facultative apomictic genotypes (Morpa, Tanganyika, Don Pablo, and Kromdraii) with different efficiencies, as the regeneration capacity is influenced by the genotype since the two sexual materials included in the study did not regenerate (Echenique et al., 1996). Later Echenique et al. (2001) analyzed four different explant sources (immature inflorescences, embryos, seeds, and leaf bases) from three out of the four genotypes previously mentioned (Morpa, Don Pablo, and Kromdraai). Immature inflorescences were the most suitable explants for inducing embryogenic calli of the three cultivars. More recently, we have started to use isolated mature embryos as explants to establish a new *in vitro* regeneration protocol. Unlike their immature counterparts, mature embryos are available in large quantities throughout the year, being easily stored in the form of dried seeds (Delporte et al., 2005). These embryos are cultured with the scutellum facing upward on MS basal supplemented medium, and they produce structures like a callus and/or somatic embryos that arise from the shoot apical meristem (SAM) and then develop seedlings. Callus formation and/or somatic embryogenesis from the scutella of immature embryos were observed in *Eragrostis tef* (Zucc.) Trotter (Gugsa and Kumlehn, 2011).

Some efforts have also been made to develop a genetic transformation protocol in this genus. Ncanana et al. (2005) reported the development of plant regeneration and transformation protocols for *E. curvula* cv. Ermelo. Calli were generated from leaf and seed tissues and were transformed by biolistic bombardment with the yeast *Saccharomyces cerevisiaeHsp12* gene under the maize ubiquitin promoter. Although successful transformation and transcription of the *Hsp12* gene occurred, no *Hsp12* protein was found in the transformed plants. Diaz (2006) evaluated the transient expression of the *uidA* gene introduced by biolistic bombardment in embryogenic calli obtained from immature inflorescences. Three different promoters were analyzed, and the maize ubiquitin gene promoter gave the best response. It was also possible to get transient transformation by inoculating mature seeds with two *Agrobacterium tumefaciens* strains, AGL0 and AGL1, both containing the binary vector Ppzp201BUGI (Terenti Romero, 2015).

Another member of the genus is *Eragrostis tef* (Zucc.) Trotter, a staple food crop in Ethiopia and cultivated in several countries for grain and forage production. It is not an apomictic species but it can also grow on marginal soils and under climate conditions not suitable for major cereals such as maize, wheat, and rice. It is an extremely low-yielding crop, mainly due to lodging and prolonged drought during the growing season (Numan et al., 2021). To increase the yield by introducing dwarfism, Gebre et al. (2013) transformed embryogenic calli obtained from immature embryos with the gibberellic acid (GA) inactivating gene *PcGA2ox* under the control of a triple CaMV 35S promoter using *Agrobacterium*. Calli were induced in a culture medium supplemented with KBP minerals and 2,4-D. The selection was performed in KBP medium with kanamycin, and regeneration in K4NB, where fully viable transformed plants carrying the transgene were obtained (detected by PCR and then sequenced). Some of them showed the expected phenotype. According to Numan et al. (2021), there must be a well-established transformation and regeneration system in *E. tef* to adopt advanced genetic engineering technologies. One of the major obstacles for the targeted breeding of *E. tef* is the presence of genes in two genomes (AA and BB: 2*n* = 4x = 40 chromosomes), as was shown in the recently released genome assembly (VanBuren et al., 2020). Target genes affecting key traits for this crop that can be modified by CRISPR-Cas have recently been selected from the genome assembly (Numan et al., 2021).

### *Panicum* Linnaeus

*Panicum* is a genus with approximately 450 species that grows in tropical and subtropical regions (Zuloaga and Soderstrom, 1985). The genus includes important forage species, such as Guineagrass (*Panicum maximum* Jacq.) and Switchgrass (*Panicum virgatum* L.), the former reproducing by apomixis. Attention to this group was particularly focused on Switchgrass owing to its inclusion in developmental programs as a model to produce lignocellulosic ethanol or second-generation biofuels (Merrick and Fei, 2015; Lin et al., 2017). It is self-incompatible and has varied ploidy levels with tetraploid and octoploid being the most common. As a perennial grass with a deep root system, Switchgrass is effective for soil conservation and could be helpful for removing the herbicide Atrazine, which is used in field crops for broadleaf weed control. In addition, this crop presents pest- and disease-tolerance traits has a high biomass yielding potential, and the ability to grow in marginal lands with low nutrient requirements (Lin et al., 2017).

Seo et al. (2010) reported an optimized protocol for shoot regeneration in these grasses starting from mature seed-derived calli of two species, *P. longijubatum*, and *P. meyerianum*. The effects of different carbohydrates and the optimal hormonal combination in the culture medium were determined. The maximum shoot regeneration was obtained using maltose (30 g/L; a twofold increase compared with sucrose) and TDZ (1 mg/L) without naphthalene acetic acid (NAA). This optimal regeneration medium was used on nine *Panicum* spp., and the efficiency of shoot regeneration resulted in being genotype-dependent, ranging from 69.9% in *P. meyerianum* to 0% in *Panicum maximum.* However, using calli derived from immature embryos in spite of mature seeds, higher regeneration frequencies (41–75%) were observed for three cultivars of Guineagrass, showing the influence of the explant source on shoot regeneration. The high capacity of immature embryos to regenerate shoots in plant tissue culture has also been reported previously in barley (Chang et al., 2003) and maize (Frame et al., 2000).

Genetic transformation has been an important tool for studying gene function and for germplasm improvement in Switchgrass. For instance, the downregulation of the cinnamyl alcohol dehydrogenase (*CAD*) gene or the caffeic acid O-methyltransferase *(COMT)* gene decreased lignin content and improved sugar release, giving Switchgrass the potential to increase digestibility and biofuel production during the fermentation processes (Fu et al., 2011a, b). In this context, several research projects have been focused on overcoming the recalcitrance of some cultivars to genetic transformation. The first report on obtaining highly regenerable type II calli used inflorescences as explants and a modified NB-based medium (LP9), containing 2,4-D and 100 mg/L^–1^ of L-proline (Burris et al., 2009). The maximum transformation efficiency (TE: transgenic plants obtained/total of calli exposed to *Agrobacterium*) of 4.4% was obtained using the EHA105 *Agrobacterium* strain. However, type II and type I calli were not separated during the transformation process and only type II callus yielded transgenic plants, suggesting that this TE can probably be increased by only selecting type II calli for transformation. Afterward, the identification and selection of type II calli from mature caryopses resulted in transformation efficiency increases of 50–90% in three different cultivars using Murashige and Skoog (1962) based medium for all the culture stages, supplemented with 2g/L^–1^ of L-proline (Li and Qu, 2011). In this study, the regeneration rate was higher than 80% in all cultivars, also showing the high competence of type II calli for the regeneration process. Importantly, vacuum application during *Agrobacterium* infection, desiccation at the co-cultivation stage, and resting after infection also facilitated Switchgrass transformation and selection (Li and Qu, 2011; Lin et al., 2017). On the other hand, histological analysis of calli derived from seeds revealed the occurrence of a “shell-core” structure being the “core” that was able to develop on type II calli (Liu et al., 2015; Lin et al., 2017). Following the dissection of this “core,” it was possible to recover transgenic plants of a cultivar previously reported as highly recalcitrant (Liu et al., 2015). In addition, it has been noticed that optimization of the seed sterilization step increased callus induction by 20% (Lin et al., 2017). Finally, the development of embryogenic cell suspension cultures derived from type II calli accelerated the regeneration of transgenic plants (Ondzighi-Assoume et al., 2019). However, somaclonal variation has been reported to occur during embryogenic callus suspension culture (Lu et al., 2006; Liu et al., 2018).

### *Pennisetum* Richard

This genus includes the apomictic *Pennisetum squamulatum* and also the sexual relatives, pearl millet (*Pennisetum glaucum* L.), and Napier grass (*Pennisetum purpureum* Schumach.). The latter two are important forage crops in tropical and subtropical areas of the world, pearl millet also being a staple food in Africa and India, and Napier grass having great potential as an energy crop, and especially as a source for biofuel production. Thus, the development of a reliable transformation protocol for these crops will complement the classical breeding programs, and the effect of putative apomixis gene(s) from *P. squamulatum* could also be tested. Apomixis in *P. squamulatum* is transmitted by the apospory-specific genomic region (ASGR), a physically large, hemizygous, and non-recombining chromosomal region (Ozias-Akins et al., 1998). Multiple copies of the *PsASGR-BABY BOOM-like* (*PsASGR-BBML*) gene were found within the ASGR, and it has been proposed as a candidate gene related to parthenogenesis. *BBM* transcripts were originally identified in microspore cultures of *Brassica napus* (*BnBBM*) undergoing somatic embryogenesis (Boutilier et al., 2002). More recently, Conner et al. (2015) attempted to analyze the function of the *PsASGR-BBML* gene in *P. squamulatum* by creating an RNAi apomictic line to knock down the expression of this gene. Since the direct transformation and regeneration of *P. squamulatum* was not possible, it was decided to clone the ASGR in the sexual tetraploid pearl millet and the transgenic plants obtained exhibited parthenogenesis (Conner et al., 2015). In addition, sexual lines with an RNAi construct to silence *BBML* genes were generated and then these lines were fertilized with pollen from *P. squamulatum.* The resulting F1 hybrids inheriting the RNAi construct and harboring the ASGR showed a reduced number of parthenogenetic embryo sacs owing to the silencing of the *BBML* gene (Conner et al., 2015). The protocol employed above for the genetic transformation of pearl millet was previously described by Goldman et al. (2003), who optimized the conditions for biolistic-mediated transformation using embryogenic callus derived from inflorescences as explants. Shaving the spikelet primordia of a single inflorescence proved to be an excellent way to increase the amount of tissue capable of embryogenic calli production. By rotating the axis of the rachis, inflorescences could be shaved multiple times, allowing large quantities of responsive spikelets. Callus induction was performed in the dark in an MS medium containing sucrose (30 g/L) and a maximum of 5 mg/l 2,4-D. For plant regeneration, BAP (0.1 mg/L) and TDZ (0.1 mg/L) were used. TDZ was beneficial due to reducing *in vitro* culture and the overall time required for regenerating plants (Goldman et al., 2003). Delaying herbicide selection by 10–14 days after transformation resulted in greater effectiveness in the recovery of resistant plants.

Herbicide selection could either allow the regeneration of escapes (even at high selection pressure) or be deleterious to the regeneration process. It is commonly observed that the necrotic tissue surrounding the positive transgenic cells may prevent their growth. However, mannose-positive selection promoted regeneration and growth of the transgenic cells while non-transgenic cells were starved but not killed (O’Kennedy et al., 2004). Transgenic phosphomannose isomerase (PMI)-expressing cells acquired the ability to convert mannose-6-phosphate to fructose-6-phosphate, whereas the non-transgenic cells accumulated the former (Reed et al., 2001). The accumulation of mannose-6-phosphate in cells inhibits phosphoglucose isomerase, thereby causing a block in glycolysis (Goldsworthy and Street, 1965). The *manA* gene was shown to be a superior selectable marker in maize and wheat when compared with antibiotic or herbicide marker genes (Reed et al., 2001; Wright et al., 2001). The *manA* gene from *E. coli* has been used to produce transgenic plants of *P. glaucum* (O’Kennedy et al., 2004). With this purpose, embryogenic calli derived from immature zygotic embryos were bombarded and regenerated in a medium with IAA (0.2 mg/L), kinetin (0.5 mg/L), and AgNO_3_ (10 mg/L). Although the transformation efficiency was low, the system was effective in selecting almost only transgenic tissue, eliminating the labor-intensive tissue culture selection and molecular analysis of putative transgenic plants (O’Kennedy et al., 2004).

Transformation of shoot apical meristems (SAMs) either through *Agrobacterium* or biolistics has already been reported (McCabe et al., 1988; Gould and Magallanes-Cedeno, 1998; Zapata et al., 1999; Cho et al., 2003; Goldman et al., 2003; Zhang et al., 2010). In the absence of tissue dedifferentiation steps, less somaclonal variation and genotype regeneration dependence were observed. Nevertheless, the direct regeneration of transformants in this method always generates chimeric plants. It was proposed that more stable transformants could be generated by the multiplication of transgenic apical meristem cells by treatment with the phenylurea-type cytokinin TDZ (Zhong et al., 1996; Srivatanakul et al., 2000; Goldman et al., 2003; Gairi and Rashid, 2004). Kinetin was also reported to induce multiple shoots in pearl millet (Jha et al., 2009), and the addition of 50 μM CuSO_4_ could enhance their proliferation in Napiergrass (Umami et al., 2012). Following this method, Jha et al. (2011) generated a multiple shoot regeneration system in *P. glaucum* (without an intervening callus phase) for *Agrobacterium*-mediated transformation. Briefly, emerging shoot apices consisting of SAMs were cocultivated with the *Agrobacterium* EHA105 strain for 3 days in an MS medium containing acetosyringone (400 μM, essential for the successful transformation of pearl millet). After the recovery phase, shoot apices were transferred to the selection medium to allow the growth of the transformed shoots. The surviving shoots were transferred onto a preregeneration medium with BAP (17.6 μM) for 4–6 weeks to stimulate the production of transgenic multiple shoots that were then separated individually and regenerated on a medium with no growth regulators for 2–3 weeks. Stable transgenics were obtained, and the highest transformation frequency observed was 5.8% (number of transgenic plants/total number of explants inoculated) (Jha et al., 2011). Previously, Yookongkaew et al. (2007) reported a related protocol for the transformation and regeneration of several Thai rice cultivars showing that this technique is efficient and genotype-independent. This technique is also an alternative for species that are recalcitrant in producing embryogenic calli and it was used efficiently for *P. purpureum*. However, after bombardment and selection, the transformation efficiency was very low (0.64%) because the regeneration capacity was compromised by the aged callus (4–12 months) (Gondo et al., 2017).

### *Poa* Linneaus

Kentucky Bluegrass (*Poa pratensis* L.) is aC3turf grass adapted to a wide range of soils and climates (Van Wijk, 1997). It is sensitive to high salinity and drought (McDonnell and Conger, 1984) and reproduces by facultative apomixis (Bashaw, 1980; Albertini et al., 2001). Gao et al. (2006) reported a protocol that made it possible to obtain a large number of transgenic plants *via* particle bombardment of embryogenic calli derived from immature embryos. Callus induction was performed in an MS-based medium supplemented with 2,4-D (2 mg/L), BAP (0.1 mg/L), and gelrite as a gelling agent. Kinetin (0.2 mg/L) was added to the regeneration medium. In this study, the efficiency of two selectable marker genes, *hpt*, and *bar*, was tested and compared during the obtention of transgenic lines. No escapes were found under selection with 100 mg/L of hygromycin or 2 mg/L of bialaphos, hygromycin being superior to bialaphos since 77.8% of the hygromycin-resistant calli regenerated plants, whereas only 34.3% of the bialaphos resistant ones were able to regenerate. Moreover, the selection of transformed calli with hygromycin was faster and required only 4–8 weeks in culture (vs. 14–16 weeks for bialaphos), and the transformation efficiency (transgenic plants/bombarded callus) was higher (TE_*Hygromycin*_ 22% and TE_*Bialapho*__*s*_7.5%). In addition, bialaphos-mediated selection resulted in the regeneration of some albino plants, probably related to the time in culture. Overall, a long *in vitro* culture period showed a decline of 68% in the regeneration capacity (Gao et al., 2006). Despite the high regeneration capacity observed in this study, it is important to mention that only one genotype was considered. Additionally, all the recovered transgenic plants showed a complex integration pattern (with rearrangements and multiple transgene insertions), showing inactivation of the *uidA* gene after 3 months in the soil, probably as a consequence of gene silencing by multiple insertions. An *Agrobacterium*-mediated protocol was described by Zhang et al. (2010) for the transformation of three Kentucky bluegrass cultivars using a multiple shoot induction approach. The *betA* gene from *E. coli* was introduced together with the *als* selectable marker gene, which encodes a choline dehydrogenase enzyme and catalyzes the synthesis of the plant stress-protectant betaine (Sakamoto and Murata, 2001). In brief, shoot apices were cultured in an MS medium supplemented with 2,4-D (0.2 mg/L) plus BAP (2 mg/L). After 20 days in culture, meristematic cell clumps were subcultured for 8–12 days in an MS medium with BAP (2 mg/L) for the formation of multiple shoot clumps (MSCs) and were then divided and proliferated for 15 days in a medium supplemented with 2,4-D (0.07 mg/L) and BAP (2 mg/L). The MSCs were then cocultured for 2–4 days with the *Agrobacterium* strain LBA4404 followed by 10 days on rest medium with cefotaxime (10 mg/L). A chlorsulfuron selection (6 mg/L) was performed for 45 days with subcultures every 15 days. The highest transformation frequency (number of PCR-positive plants/number of shoot tips evaluated) was 1.42% with 87.5% of the surviving shoots being escapes (Zhang et al., 2010).

### *Cenchrus* Linnaeus

Buffelgrass (*Cenchrus ciliaris* L. syn. *Pennisetum ciliare*) is a warm-season C4 perennial forage grass native to Africa and India, reproducing predominantly through apomixis (Fisher et al., 1954; Bhat et al., 2001). It is highly drought-tolerant and is mainly used as fodder owing to its high biomass productivity (Martin et al., 1995; Rao et al., 1996). Some cultivars have shown recalcitrance to somatic embryogenesis (Colomba et al., 2006), and it was proposed that the understanding of the developmental events during this process could help in determining the role of phytohormones during the induction of somatic embryos (Yadav et al., 2009). These authors reported a histological analysis of regenerating calli of apomictic and sexual plants obtained from mature seeds, shoot tips, and immature inflorescences. Only the latter explant was able to produce embryogenic calli (with a maximum of 33%) in an MS medium supplemented with BAP (0.5 mg/L) and 2,4-D (with a maximum concentration of 5 mg/L) (Yadav et al., 2009). Regeneration of somatic embryos was obtained in the same medium supplemented with 2,4-D (0.25 mg/L) and BAP or kinetin (1–5 mg/L), with both cytokinins yielding comparative results. In the same way (Carloni et al., 2014), somatic embryos were induced from three apomictic genotypes of Buffel grass. Anthers were placed on MS-based induction medium containing 2,4-D (6 mg/L) for 90 days (subcultured after 45 days), and the regeneration was achieved in a MS medium supplemented with BAP (1 mg/L) and NAA (0.5 mg/L). Although anthers have been proposed as an optimal tissue for somatic embryogenesis (Kruczkowska et al., 2002), between 8 and 12 months were spent following this protocol for the regeneration. After this time several chromosomal rearrangements were found in the regenerated plants (Carloni et al., 2014).

Using a different approach, Kumar and Bhat (2012) tested the regeneration of five genotypes of Buffelgrass (including the apomictic one studied by Yadav et al., 2009) *via* multiple shoot induction. Briefly, seeds were germinated in an MS medium with TDZ (3 mg/L) to induce a high concentration of endogenous cytokinins (according to Yookongkaew et al., 2007). Shoot tips from 4-day old seedlings were excised and subcultured for 3–5 weeks with the same concentration of TDZ (3 mg/L). Shoots regenerated on medium containing TDZ (≥3 mg/L) were found to be stunted. To overcome this problem, GA_3_ (2 mg/L) was added to the shoot elongation medium. After 2 weeks, shoots were divided and transferred to MS plus indole-3-acetic acid (IAA, 3 mg/L) for rooting. All the genotypes were shown to be highly responsive to multiple shoot induction in a relatively short period of time (10–11 weeks). Based on these results, Laishram et al. (2020) performed an assessment of biolistic and *Agrobacterium*-mediated genetic transformation methods in *C. ciliaris*. Immature inflorescences of an apomictic cultivar were cultured in an MS medium supplemented with 2,4-D (3 mg/L) and BAP (0.5 mg/L) for embryogenic callus induction. Calli were maintained in an MS medium with casein hydrolyzate (0.3 g/L), L-proline (0.4 g/L), and L-glutamine (0.4 g/L) (according to Shashi, 2013), and subcultured every 21 days. It was observed that the regeneration ability declined after the third round of subcultures, and therefore earlier calli were used for genetic transformation. The marker gene *GUS* was used to screen transgenic events and hygromycin (30 mg/L) was employed during its selection. Stable transgenic plants were obtained only by biolistics with the transformation efficiency (transgenic plants confirmed by PCR/calli bombarded) of 0.2%. Although the regeneration rate was high (40 plants could be regenerated from the 100 resistant calluses obtained), there were a lot of escapes (90%), suggesting that further optimization of the transformation protocol is needed instead of the regeneration one.

### *Urochloa* (syn. *Brachiaria*) von Ledebour

*Urochloa* P. Beauv. (syn. *Brachiaria* Griseb. spp.) species are tropical forage grasses native to Africa (Keller-Grein et al., 1996), well adapted to poor soils, and resistant to long dry periods (Pinheiro et al., 2000). These grasses were introduced into South America in the eighteenth century (Miles et al., 1996), and are cultivated in millions of hectares in Brazil for cattle grazing (Montanari, 2013). Most of this cultivated area is covered by only two cultivars, both apomictic and tetraploid, *U. brizantha* cv. Marandu and *U. decumbens* cv. Basilisk (Simioni and Valle, 2009). Another species, *U. humidicola*, also apomictic and usually hexaploid, was introduced into Brazil from Australia. The only cultivated diploid and sexual species is *U. ruziziensis*, native to the African savannas (Valle and Savidan, 1996). For the latter species (*U. ruziziensis*), Ishigaki et al. (2009) established protocols for the development of MSCs and embryogenic calli using SAMs as explants. The addition of 2,4-D (0.5 mg/L) and BAP (2 mg/L) to an MS medium was the most effective treatment for the development of MSCs (21.4%). The highest regeneration capacity (53.6%) was observed in the same basal medium supplemented with BAP (1 mg/L) or with kinetin and GA_3_ (2 mg/L both). The highest rate of embryogenic callus induction (16.7%) was recorded in an MS medium with the addition of 2,4-D (4 mg/L) and BAP (0.2 mg/L), whereas the most effective treatment for plant regeneration was the use of solid MS medium supplemented with BAP (2 mg/L) and NAA (0.1 mg/L) (47.6%). MSCs and calli were maintained by subculturing onto fresh media every 30 or 14–21 days, respectively. Embryogenic calli maintained its shoot regeneration capacity for more than 1 year. Although both MSCs and embryogenic calli showed a high regenerative potential, some albino plants were obtained from the calli (Ishigaki et al., 2009). Afterward, in a transient GUS expression assay, Ishigaki et al. (2012) determined that embryogenic calli were more suitable targets than MSCs for particle bombardment transformation. These authors bombarded 9-month-old embryogenic calli obtaining four bialaphos resistant, but sterile, plants (TE: 1.4%). Later, by using flow cytometry it was observed that embryogenic calli older than 2 months were polyploydized and these events progressed over time (Ishigaki et al., 2014). The authors hypothesized that this response in the culture could be triggered by the accumulation of 2,4-D in old calli.

Cabral et al. (2011) evaluated the natural tetraploid apomictic genotype *U.brizantha* cv. Marandu and the diploid sexual *U. brizantha* for their capacities to produce somatic embryos and regenerate plants. When mature seeds of the apomictic cv. were used as explants, the highest induction of embryogenic calli (77%) and plant regeneration (54%) were observed in MS media with 2,4-D (3 mg/L) and BAP (0.2 mg/L) or BAP (1 mg/L), NAA (0.5 mg/L), and kinetin (2.5 mg/L), respectively. Calli were subcultured monthly on fresh medium and maintained for 5 months, but It was observed that 4-month-old cultures only produced albino plants. Interestingly, a decrease in the pH of the medium (from 5.8 to 4.0) not only reduced contamination but also increased the number of regenerating buds/shoots per clump and the total of regenerated plants. The same basal medium and growth regulator combination gave the best results regarding somatic embryogenesis and plant regeneration using leaf basal segments as explants (Cabral et al., 2011). These authors also observed that the apomictic genotype showed higher percentages of somatic embryogenesis and regeneration (77 and 64%, respectively) than the sexual one (45 and 21%), highlighting a genotype dependent response in *U. brizantha*. Afterward, with the optimal culture conditions for somatic embryogenesis described above, embryogenic calli, and cell suspensions were bombarded with plasmids containing GUS and *hptII* cassettes and tested for transient and stable expression (Cabral et al., 2018). The phased selection on hygromycin (5–10–20 mg/L) only delivered one transgenic plant, and it was negative for GUS histochemical assay and the amplification of the *hptII* gene by PCR. On the other hand, bombarded cell suspensions produced hygromycin-resistant calli that also showed stable GUS expression (both *GUS* and *hptII* genes were detected by PCR). These calli maintained their proliferative capacity for 10 months but were not able to regenerate shoots.

Takamori et al. (2015) tested two synthetic auxins (2,4-D and picloram) at different concentrations for callus induction, somatic embryogenesis, and plant regeneration in *U. brizantha* cv. Marandu using mature seeds as explants, obtaining more regenerated plants with picloram (1 mg/L). In the same study, 2,4-D at a concentration of 8 mg/L showed the lowest shoot conversion and the production of albino plantlets (4 and 8 mg/L). No albinos were observed with picloram. The regeneration was performed initially on MS with no growth regulators after maintaining the embryogenic calli for 72 days in callus induction media (subcultured every 14 days). Indirect light (1.7 μMm^–2^s^–1^) for 14 days was essential to avoid purple pigmentation (anthocyanin production) in regenerating calli. Finally, the calli were transferred to MS (half-strength) supplemented with BAP (2 mg/L) for 14 days. The effect of picloram was also tested for the embryogenic potential of other *Urochloa* species (*U. decumbens*, *U. humidicola*, and *U. ruziziensis*) with 1 mg/L being the most effective concentration. *U. brizantha*and *U. decumbens* were the genotypes that were most prone to somatic embryogenesis, whereas *U. humidicola* and *U. ruziziensis* showed a minimal response. Similar results were obtained by Yaguinuma et al. (2018) using leaf base segments as explants. In *U. decumbens*, 1 mg/L of picloram was more effective in regenerating plants than 1 mg/L of 2,4-D (49 vs. 14%), and *U. ruziziensis* tended to be more recalcitrant for plant regeneration. Furthermore, the authors proposed cytokinin (TDZ) as an alternative callus inductor to reduce somaclonal variation linked to auxins. In this way, although fewer calli were obtained, plantlets of all genotypes were regenerated with 4 mg/L of TDZ, but the best response came from *U. brizantha.* Finally, picloram also showed better results than 2,4-D in a transient GUS expression experiment.

### *Paspalum* Linnaeus

Bahiagrass (*Paspalum notatum* Flugge) is native to South America and used as turf and forage grass in tropical and subtropical regions from the United States to Argentina (Burton, 1967). This grass tolerates marginal soil fertility and has excellent resilience against drought, heat, and insect and nematode invasion. Two cytotypes, sexual diploids and apomictic tetraploids of Bahiagrass are grown (Forbes and Burton, 1961). The apomictic tetraploid cultivar “Argentine” is superior to the sexual diploid “Pensacola” owing to its darker green color, higher density, and seed production in shorter periods. Hence, with its asexual seed production and uniform seed progeny, the “Argentine” cultivar is an attractive target for genetic engineering of stress tolerance. Altpeter and James (2005) established a transformation protocol using embryogenic calli derived from mature seeds of this cultivar that were induced in an MS medium supplemented with BAP (5 μM) and dicamba (13.5 μM). After 6 weeks in culture, the calli were bombarded with a constitutive *nptII* expression cassette. The selection was initiated 1 week later on paromomycin (50 mg/L) and performed for 4 weeks (with subcultures every 2 weeks) on an MS-based shoot regeneration medium supplemented with BAP (5 μM). With this protocol, 2–10% resistant calli were obtained. Out of these, 1.5–4% regenerated plants and 60% of these plants were confirmed by PCR to be transgenic. Based on this protocol, James et al. (2008) obtained apomictic transgenic plants of Bahiagrass expressing the transcription factor *DREB1A* from barley, regulated by the abiotic stress-inducible HVA1 promoter. Transgenic lines showed faster recovery and increased survival under repeated dehydration–rehydration cycles than wild-type plants. Moreover, the transgene did not affect normal plant growth under non-stress conditions. The same protocol was used for the constitutive expression of *HvWKY38* in transgenic plants of Bahiagrass (Xiong et al., 2010), obtaining a regeneration efficiency (resistant calli/calli bombarded) of 3.7% and a TE (transgenic lines/calli bombarded) of 2.7%. Although some lines were sterile and others had reduced seed set, three lines (out of 17 obtained) showed wild-type phenotypes and produced a similar number of inflorescences and seeds. The transgenic plants showed improved survival and biomass accumulation following dehydration and were superior to the wild-type plants in water retention, regeneration of new roots, and photosynthetic efficiency.

Uniform transgene transmission and expression could be a challenge during biolistic-mediated transformation, especially when large amounts of DNA are delivered into cells. Furthermore, transgenic plants frequently show complex integration patterns and silencing of transgenes (Gao et al., 2006). For this reason, Sandhu et al. (2007) attempted to optimize the process using two minimal transgene expression cassettes for particle bombardment-mediated cotransformation in Bahiagrass. Two unlinked *nptII*and *bar* genes were excised from plasmids and cointroduced into embryogenic calli derived from mature seeds of the apomictic cv “Argentine.” After selection on medium containing paramomycin as selection agent, a TE of 10% was obtained with 95% of cotransformation frequency. *Bar* gene showed more complex integration patterns, probably owing to the presence of recombination hotspots at the CaMV35S sequence. The presence of recombinogenic spots within the expression cassettes and larger quantities of DNA during transformation seems to increase the complexity of transgenic loci (Sandhu and Altpeter, 2008). Despite the complex integration of these minimal cassettes, high expression, and coexpression levels were observed. Probably, this could be due to insertions on different loci whereas plasmid sequences tend to become linked. With a similar approach, Agharkar et al. (2007) achieved the constitutive expression of the *AtGA2* gene in Bahiagrass (cultivar “Argentine”). Minimal cassettes of *nptII* and *AtGA2* were transferred through biolistics and it resulted in 100% of cointegration and coexpression together with a simple integration pattern. No escapes were observed during the selection process and a TE of 1.3% was reached (eight independent transgenic lines were obtained from 600 bombarded calli). The *AtGA2* expressing lines hydroxylated and degraded the GAs showing very low levels of GA, which was reflected in shorter stems and leaves, delayed flowering, and an increase in tillering. So, the semidwarf phenotype showed an enhanced turf quality.

Antibiotic selection through the constitutive expression of gene-conferring resistance is not always desirable in plant genetic engineering because it may hamper the normal growth or recovery of transgenic plants. The use of a visual marker without chemical dependence has made it possible to increase the transformation efficiency and reduce the screening time (Ahlandsberg et al., 1999; Zhu et al., 2004; Baranski et al., 2006). Accordingly, Himuro et al. (2009) proposed visual screening and selection of transgenic diploid Bahiagrass plants using the *GFP* reporter gene. Embryogenic calli for particle-bombardment transformation were induced from mature seeds in a liquid MS medium supplemented with 2,4-D (2 mg/L). After 2 weeks, primary calli were transferred to solid MS with the same concentration of 2,4-D. Then, microcalli (not larger than 2 mm in diameter) were divided and subcultured every 2 weeks in a medium containing 2,4-D (2 mg/L), BAP (0.1 mg/L), and CuSO_4_ (50 μM) at 31°C. Microcalli were transformed and the GFP expressing sectors were separated using fine-tipped forceps and transferred to a fresh medium. All these visually selected calli were able to regenerate in an MS medium with GA_3_ (1 mg/L) and kinetin (1 mg/L), obtaining an overall TE of 2.4%. Microcalli production resulted in the exposure of a larger surface area for the approaching particles during the bombardment and also for the culture conditions. Previously, it had been reported that high Cu content and high temperature during *in vitro* culture could probably enhance the regeneration of transgenic plants (Gondo et al., 2005). Furthermore, these conditions were adopted by Mancini et al. (2014) who observed a regeneration efficiency of 78% during the genetic transformation of tetraploid apomictic Bahiagrass. In this study, mature seeds were used as explants for embryogenic calli induction in an MS medium with B5 vitamins and 2,4-D (0.25 mg/L), and calli were cotransformed with plasmids containing *egfp* and *bar* genes. Regeneration was performed in an MS medium with B5 vitamins supplemented with BAP (5 μM), GA_3_ (1 μM), and CuSO_4_ (50 μM). A cotransformation frequency of 40.7% and a TE (independent transgenic lines/calli bombarded) of 8% were observed. The transgenic plants obtained were phenotypically normal and fertile after 4 weeks of selection in ammonium glufosinate (1 mg/L). Afterward, this protocol was successfully applied in *Paspalum notatum* for the functional characterization of the apomixis-related genes *QUI-GON JINN (QGJ)* and *trimethylguanosine synthase 1* (*TGS1)*-like (Mancini et al., 2018; Colono et al., 2019). The high Cu content in the culture medium as a regeneration enhancer, firstly reported by Gondo et al. (2005) and later validated, appears to be a promising approach that could be replicated in other warm-season recalcitrant species. On the other hand, Giordano et al. (2014) obtained transgenic *Paspalum dilatatum* plants in which a sense-suppression gene cassette, delivered free of vector backbone and integrated separately to the selectable marker, reduced the *CCR1* transcript levels and, as a consequence, the lignin content.

## Significant Breakthroughs in Grass Transformation

### Alternative DNA-Delivery Methods

The success of the induction of embryogenic callus, somatic embryos, and the resulting recovery of viable plants is not always achievable for many species. Furthermore, tissue culture may demand long and complex treatments or procedures, and it often causes undesired and unpredictable changes in plant genomes. The *in planta* transformation methods avoid callus culture and plant regeneration (Figures 1B,C). The most widely used of these methods is a floral dip, originally developed for *Arabidopsis* (Clough and Bent, 1998), where the flower buds are dipped into an *Agrobacterium* suspension leading to the transformation of the ovule cells (Figure 1B). Floral dip methods have also been described for grass species including wheat (Zale et al., 2009), maize (Mu et al., 2012), *Setariaviridis* (Martins et al., 2015; Saha and Blumwald, 2016), and rice (Ratanasut et al., 2017). However, a broader application remains elusive mainly because the method is dependent not only on successful *Agrobacterium* infection but also on the inflorescence anatomy and the capacity of the plant to produce large amounts of seeds. Direct DNA delivery into pollen cells by ultrasonication or magnetofection has also been described as an alternative to creating transgenic seeds through pollination of plants with transformed pollen (Yang et al., 2017; Zhao et al., 2017). In addition to gametophyte cells, SAMs were used as targets for *in planta* biolistic transformation in wheat (Hamada et al., 2017; Figure 1C). Although primary T_0_ plants were chimeric, it was possible to recover T_1_ plants carrying the transgene, suggesting that genome modifications occurring at the SAMs are able to pass to the next generation. Even when the transformation efficiency (transgenic T_1_ plants/embryos bombarded) was lower than that of conventional tissue culture-based methods (0.8%), stable transformed lines of the recalcitrant cultivar Haruyokoi were obtained.

With regard to the limited host range of *Agrobacterium tumefaciens*, the compelling need to identify or generate non-*Agrobacterium* delivery systems emerges to increase the scope of genetic transformation. Furthermore, *Agrobacterium*-engineered crops for commercialization are restricted by licensing (Chi-Ham et al., 2012). It has long been recognized that the machinery required for DNA delivery to plant cells is not exclusive of *Agrobacterium*, since it is also present in species of the *Rhizobium* genus (Broothaerts et al., 2005; Rudder et al., 2014; Lacroix et al., 2016; Lacroix and Citovsky, 2019). It was observed that *R. etli* can successfully transform *N. benthamiana* and *N. tabacum* with a plasmid containing a T-DNA segment (Lacroix and Citovsky, 2016). However, the expression levels are about 10 times lower than those obtained with *A. tumefaciens.* Nonetheless, *R. etli* might be more efficient with other plant species, such as its specific hosts. Alternatively, Zuniga-Soto et al. (2015) obtained transgenic rice with *Ensifer adhaerens-*mediated infection of two Japonica cultivars with transformation efficiencies comparable with the *Agrobacterium-*mediated ones. Moreover, with this method, these authors were able to obtain a transgenic line from the recalcitrant Indica variety IR64. The genome of *E. adhaerens* (syn. *Sinorhizobium adherens)* not only has the same essential genes as *Agrobacterium* T-DNA but also non-essential ones, which could exert a positive impact on the virulence and ability to transform host tissues (Rudder et al., 2014). Although *E. adharens*-based protocols are still at the early stages, there are sustained efforts to expand them (Rathore et al., 2019) and indeed, they have a high potential in crop biotechnology.

On the other hand, virus-based vectors provide an alternative for delivering genome engineering reagents to plant cells (Liu and Zhang, 2020; Wang et al., 2020). Among these are the RNA viruses, which for monocots include wheat streak mosaic virus (WSMV) and barley stripe mosaic virus (BSMV) (Lee et al., 2012). Single-stranded (ss) DNA viruses, such as geminiviruses, are able to infect a wide range of host plant species like wheat, barley, corn, oat, and rye, and have been widely adopted as vectors (Choi et al., 2000). Furthermore, they replicate inside the host cells and produce a high amount of replicons enhancing the targeting cell response (Hanley-Bowdoin et al., 2013). Although the cargo capacity of geminiviruses is quite restricted, these vectors have been engineered for the expression of heterologous proteins in plants (Lozano-Durán, 2016). BSMV-based vectors were used for gene silencing in barley (*Hordeum vulgare*) and could be a suitable option in grasses as well as the RNAi approach (Holzberg et al., 2002; Scofield and Nelson, 2009). The virus-induced gene silencing exploits the RNA-based plant antiviral defense response, which degrades the RNA produced by infecting viruses. In this way, by inserting a plant gene sequence into the viral vector, gene transcripts become targets for degradation (Kumagai et al., 1995; Ratcliff et al., 1997; Baulcombe, 1999).

Genome editing (GE) is a new powerful technique and is considered a precision plant breeding tool (Kausch et al., 2019). Thus, the CRISPR/Cas technology used in its multiple versions (*via* transgenesis or by delivering ribonucleoproteins, base, and prime editing, etc.) offers new opportunities for improving different traits in important crops, sometimes combined with synthetic biology (for a review, see Chen et al., 2019; Zhu et al., 2020). Furthermore, nanotechnology has shown higher transformation efficiency than conventional methods, and in combination with GE, it has a great potential for engineering plants (Ahmar et al., 2021). The above considerations that influence the response of grasses concerning tissue culture, plant regeneration, and plant transformation methods are also important for GE. The knowledge generated from functional studies in apomictic grasses through transgenesis could support the induction of apomixis *via* GE in sexual plants to preserve the hybrid vigor for multiple generations in economically important crops (Scheben and Hojsgaard, 2020; Fiaz et al., 2021). However, the genomic information in apomictic grasses needed to perform GE is scarce and often unavailable. Although no genome-edited apomictic grasses have yet been reported, targeted mutations have been achieved in related grasses, such as *Panicum virgatum* (Liu et al., 2018) and *Setariaviridis* (Basso et al., 2021), using the CRISPR/Cas technology.

### Agrobacterium–Host Interaction

To exploit *Agrobacterium* as a routine biological engineering tool, it was necessary to develop the “binary vector” system from the large tumor inducing (Ti) plasmid (Hoekema et al., 1983; Bevan, 1984). This system allowed the separation of transfer DNA (T-DNA) in a small episome from the virulence genes encoded by a disarmed Ti plasmid. Afterward, the superbinary vector pSB1 with extra *vir* genes (B, G, part of C and D) was developed and resulted in the expansion of the host range of plants amenable to transformation (Komari, 1990; Hiei et al., 1994; Ishida et al., 1996; Komari et al., 1996; Cheng et al., 1997; Tingay et al., 1997). Recently, an improved pVIR ternary vector system was able to enhance the transformation frequencies of recalcitrant maize inbreds (Anand et al., 2018). Moreover, transformed calli grow faster, reducing the transformation process by 2–3 weeks. However, a high non-quality events frequency (i.e., events with backbone integration and/or multicopy events) was observed, probably due to the increased T-strand delivery. Similar results were described using the hypervirulent strain of *Agrobacterium* AGL0 (Zhi et al., 2015).

*Agrobacterium* elicits a wide plant defense response and modifies cell metabolism during infection, which in turn could affect the transformation efficiency (Ditt et al., 2006; Torres et al., 2006; Zipfel et al., 2006). *Myo*-inositol has several functions in eukaryotes, affecting a variety of developmental and physiological processes (Boss et al., 2006; York, 2006; Michell, 2008; Munnik and Nielsen, 2011), and it is a common constituent of standard plant culture media, as its addition is believed to improve plant regeneration (Murashige and Skoog, 1962; Gamborg et al., 1968; Loewus and Loewus, 1983; Schenk and Hildebrandt, 2011). However, a report in Ryegrass (*Lolium perenne* L.) revealed that the removal of *myo*-inositol from the callus culture media, in combination with a cold shock pretreatment and the addition of L-glutamine (L-Gln) prior to and during *Agrobacterium*-infection, resulted in about 84% of the treated calli being stably transformed (Zhang et al., 2013). Additionally, nearly 60% of the stable-transformed calli regenerated into green plants. Furthermore, the authors showed that under these conditions, the expression of pathogenesis-related (PRs) genes is modified, and probably plant defense response is attenuated, leading to an enhancement in the transformation efficiency. Similar results were obtained when the protocol was applied to cells, a rice cells suspension, suggesting that a broad application of this protocol can improve the transformation of recalcitrant monocot species (Zhang et al., 2013).

### Gene Modulation in Target Cells

An alternative way to improve transformation and/or minimize callus culture concerns in grasses emerges from a genetic perspective. The ectopic expression of a few transcription factors (TFs) can induce the development of spontaneous somatic embryos in transformed tissues (Lotan et al., 1998; Stone et al., 2001; Boutilier et al., 2002; Figure 1D). Studies on its role in 2,4-D-mediated somatic embryogenesis have revealed an interacting network that acts on hormone pathways (see Horstman et al., 2017 and reference within). In the light of these facts, Lowe et al. (2016) successfully enhanced somatic embryogenesis and transformation in recalcitrant maize, rice, sorghum, and sugarcane varieties through the constitutive coexpression of TFs as *BBM* and *WUS2*. In this report, immature embryos were directly transformed by *Agrobacterium*-mediated infection, resulting in growth stimulation of embryogenic tissues. However, once embryogenic callus is formed the *BBM* and *WUS2* expression cassettes must be eliminated to avoid deleterious pleiotropic effects in the regenerating plants. Although the excision method employed was efficient in the recovery of healthy and fertile, male and female plants, 3 months of calli culture was still required. An alternative scenario was described later by restricting the expression of *BBM* and *WUS2* to the transformation targets (scutella and young leaves) using specific regulatory elements (Lowe et al., 2018). In this way, somatic embryos were developed on immature embryos within the first week after *Agrobacterium* infection. Furthermore, these embryos were able to germinate into healthy fertile plants without any intervening callus phase. This direct somatic embryogenesis response appeared to be genotype independent, and often dozens of somatic embryos could be observed on the surface of the developing scutella at similar rates as that of the zygotic embryos. This method requires less than half the normal time for traditional callus-based transformation protocols and eliminates the somaclonal issue. In another genetic approach, Debernardi et al. (2020) demonstrated that the overexpression in calli of a sequence encoding a chimera, composed of the *GRF4 (growth-regulating factor 4)* transcription factor and its *GIF1 (GRF-interacting factor 1)* cofactor, substantially increased the regeneration efficiency in wheat, rice, and citrus, including recalcitrant species. Also, the *GRF4–GIF1* chimera resulted in high transformation and regeneration efficiencies, even in shorter protocols and contingent upon diverse conditions. On this basis, a protocol to select transgenic shoots without using antibiotic-based markers was formulated with promising results. The addition of the *GRF4–GIF1* chimera in wheat also allowed the regeneration of transgenic lines in a cytokinin-free medium. Furthermore, transgene excision was not necessary to recover normal and fertile plants.

## Final Remarks

Owing to the efficient use of energy and the high adaptability to harsh conditions, apomictic grasses, many of which are C4, constitute a source of genes that can be useful for improving agronomic traits in crop plants, such as high yield and resistance to biotic and abiotic stresses, especially in the current climate change scenario. Also, this group of grasses represents very useful resources either for second-generation biofuel production or forage. Furthermore, transferring apomixis into economically important crops would be one of the major achievements of modern agriculture. A prerequisite to doing this is to understand how apomixis works. The way to acquire this knowledge is by modifying candidate genes directly in apomictic species that provide the correct epigenetic landscape for the expression of these genes. In this way, it would be possible to characterize their correct function. However, an efficient transformation protocol is still needed to engineer these grasses. Recently, several advances that contributed to enhancing the embryogenic and regeneration responses of cultured tissues have been obtained. Among these, high Cu content, the use of maltose instead of sucrose, a decrease in pH (from 5.8 to 4.0), and the removal of myo-inositol from the culture media have shown to be effective for enhancing the regeneration response. In addition, the use of alternative auxins and the expression of morphogenic genes during *in vitro* culture, the *in planta* genetic transformation methods, and genome editing emerge as promising approaches to increase the transformation efficiency of apomictic grasses.

## Author Contributions

AB and VE: conceptualization and writing original draft. VE: supervision. AB, ES, HP, and VE: writing, review, and editing. All authors have read and agreed to the published version of the manuscript.

## Conflict of Interest

The authors declare that the research was conducted in the absence of any commercial or financial relationships that could be construed as a potential conflict of interest.

## Publisher’s Note

All claims expressed in this article are solely those of the authors and do not necessarily represent those of their affiliated organizations, or those of the publisher, the editors and the reviewers. Any product that may be evaluated in this article, or claim that may be made by its manufacturer, is not guaranteed or endorsed by the publisher.

## Abbreviations

2,4-D: 2,4-dichlorophenoxiyacetic acid
BAP: 6-benzylaminopurine
Dicamba: 3,6-Dichloro-2-methoxybenzoic acid
GA: Gibberellic acid
IAA: Indole acetic acid
NAA: Naphthalene acetic acid
TE: Transformation efficiency
TDZ: Thidiazuron.
